# Repair of isolated horizontal meniscal tears with all-inside suture materials using the overlock method: outcome study with a minimum 2-year follow-up

**DOI:** 10.1186/s13018-016-0466-y

**Published:** 2016-10-28

**Authors:** Uğur TİFTİKÇİ, Sancar SERBEST

**Affiliations:** Department of Orthopaedics and Traumatology, Faculty of Medicine, Kırıkkale University, Kırıkkale, Turkey

**Keywords:** Meniscus, Horizontal tear, Degenerative tear, Meniscus repair

## Abstract

**Background:**

This study aimed to consider the use of a meniscal repair in patients in order to close the horizontal cleavage extending up to the avascular zone. The hypothesis was to examine the clinical and arthroscopic outcomes following meniscal repair of degenerative horizontal cleavage tears with new-generation all-inside suture materials using the overlock method.

**Methods:**

We retrospectively reviewed a consecutive series of 55 patients which had a horizontal pattern, and finally, 27 patients with a horizontal tear only which required no additional intra-articular surgical intervention were included in the study. Arthroscopic meniscal repair was performed using the overlock method. Functional outcomes were evaluated using Lysholm knee scoring scale, Cincinnati scores, subjective International Knee Documentation Committee (IKDC) criteria and Tegner activity scale. Assessment of meniscal healing was evaluated clinically by the presence of meniscal signs. The preoperative and postoperative MRIs were examined.

**Results:**

The mean follow-up period was 29 months (range, 24–38). The mean Lysholm score improved significantly from 59.5 ± 12.4 points preoperatively to 90.0 ± 4.7 points postoperatively (*P <* 0.0001). The Cincinnati score increased from 14.8 ± 5.3 to 26.9 ± 1.6 (*P <* 0.0001). The Tegner activity score increased from 3.7 ± 1.4 to 6.4 ± 1.6 (*P <* 0.0001). The mean IKDC subjective score also improved significantly from 48.5 ± 9.8 preoperatively to 90.4 ± 5.0 postoperatively (*P <* 0.0001).

**Conclusion:**

Meniscal repair of degenerative horizontal tears using the overlock method resulted in improved Lysholm and IKDC subjective scores. With careful selection of the patients and the horizontal meniscus tears, the success of the meniscus repairs increases. Repair can be recommended for all horizontal meniscus tears which can be repaired arthroscopically.

## Background

Meniscus tears are the most frequently treated knee injuries [1]. The form of meniscus tears may be longitudinal, vertical, oblique, peripheral, complex, transverse, radial, and horizontal. The capacity for meniscus tears to be repaired depends on several factors, such as vascularity, chronicity, type of tear, location, and size [2]. Horizontal meniscus tears are traumatic and degenerative tears of the structure [3]. These tears are generally in the zones 3 and 4 posterior of the medial meniscus and, by progressing to the meniscocapsular region, can cause parameniscal cysts to form [4, 5]. The meniscus splits into two layers. In the majority, the inferior layer is unstable and weak. The repair of these tears is difficult as the sutures of the repair applied may not be sufficient against the mechanical pressures, and associated with this, poor healing rates have been reported [6]. Meniscectomy has been recommended for the weak meniscus layer section in horizontal tears [7].

Presently, the treatment options available for patients with horizontal degenerative cleavage tears of the meniscus are limited. These tears are considered an indication for partial or subtotal meniscectomy because when the tear is located within an avascular area, it is difficult to induce healing. However, meniscectomy is not ideal because it disrupts the normal anatomical structure and function of the meniscus. In this study, an evaluation was made of the treatment efficacy and recovery in patients after the repair of horizontal meniscus tears with new-generation all-inside suture materials using the overlock method.

## Methods

### Patients and method

**Fig. 1:**
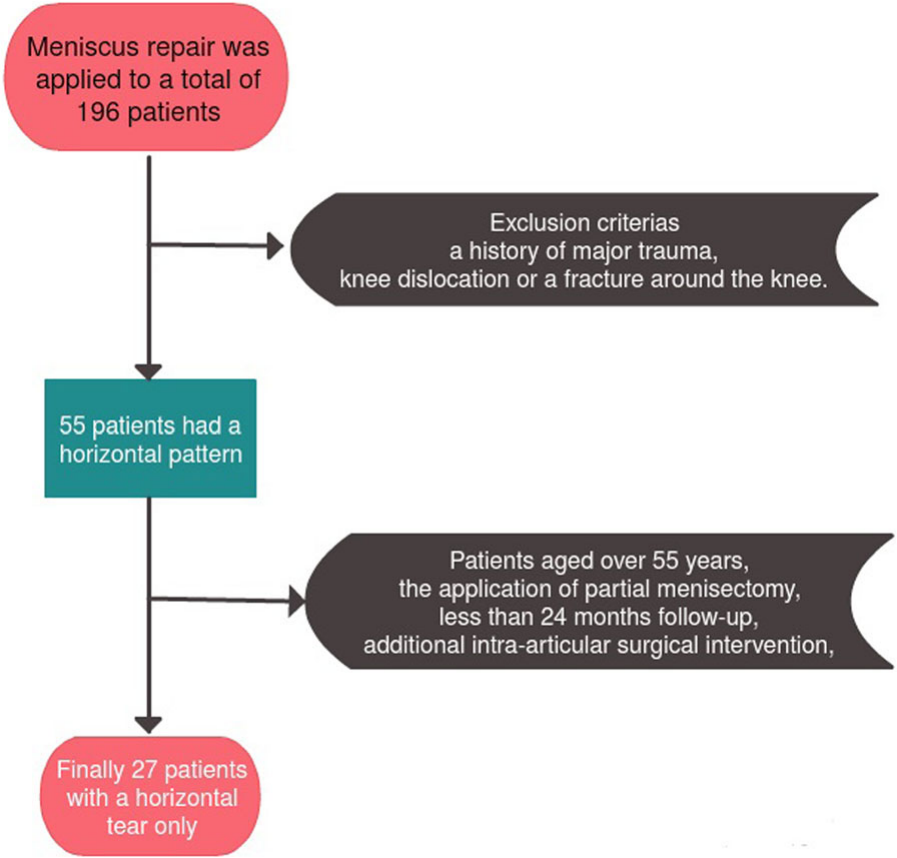
Flow diagram of exclusion criteria

**Fig. 2:**
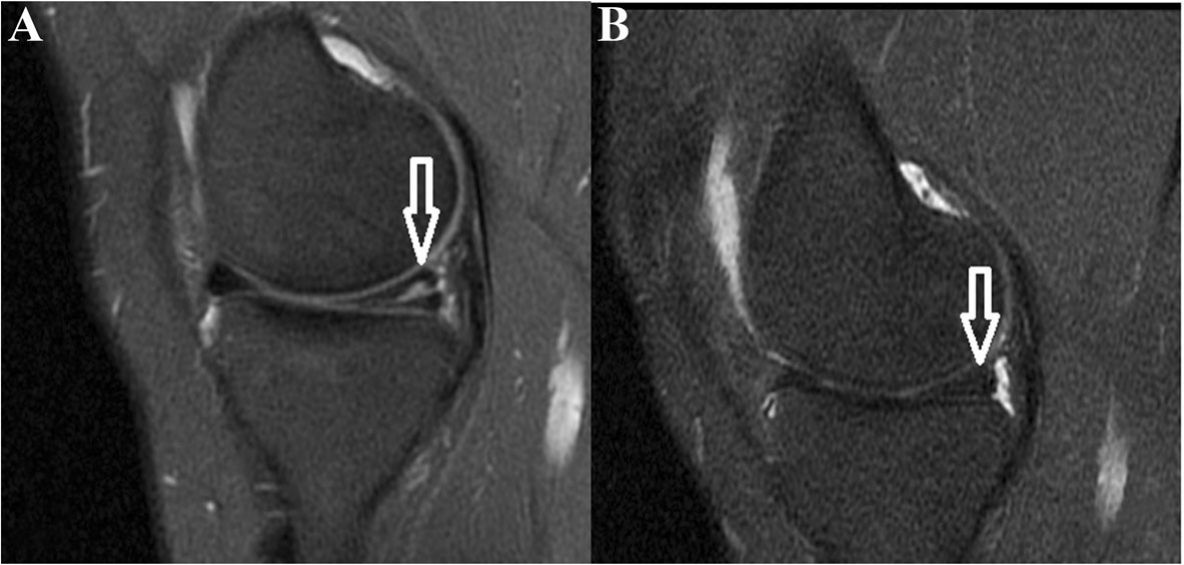
A sagittal T2-weighted MRI shows horizontal tear (*white arrow*) at the posterior horn of medial meniscus preoperatively (**a**) and postoperatively (**b**) who did not have a history of distinct previous trauma

Meniscus repair was applied to a total of 196 patients in the Orthopaedics and Traumatology Clinic between 2011 and 2014. Of these, 55 patients had a horizontal pattern, and finally, 27 patients with a horizontal tear only which required no additional intra-articular surgical intervention were included in the study. Approval for the study was granted by the Local Ethics Committee, and informed consent was obtained from all the patients (2015/10-02). The patients included in the study were aged below 55 years, have tears in the zones 3 and 4 posterior of the meniscus, were determined with a horizontal meniscus tear on MRI and during arthroscopic surgery, which was able to be repaired, and had a follow-up period of at least 24 months. Exclusion criteria were patients aged over 55 years, the application of partial meniscectomy, less than 24 months follow-up, additional intra-articular surgical intervention, a history of major trauma, knee dislocation or a fracture around the knee, ACL tear, nicotine intake, drugs, diabetes and all other systemic factors (Fig. 1). A record was made of preoperative age, affected extremity, symptoms (locking, pain, swelling), preoperative and postoperative Lysholm scores, Cincinnati scores, subjective International Knee Documentation Committee (IKDC) criteria, Tegner activity score [8], Barrett criteria [9] (sensitivity in the joint area, effusion and McMurray test) and Kellgren-Lawrence [10] grading for degenerative arthritis on the radiograph taken at the final follow-up (Table 1). The preoperative and postoperative MRIs were examined (Fig. 2).

**Table 1:** Symptom of the patients, physical examination and MRI findings

Patients (*n =* 27)		Preoperative	Postoperative 24 months
Kellgren-Lawrence grading			
Grade 0/1/2/3		20/4/3/0	20/4/3/0
Symptoms	Locking	8	0
	Pain	13	1
	Swelling	11	0
Barret criteria	McMurray test	13	1
	Joint line tenderness	15	1
	Effusion	5	0
MRI findings			
Grade 1/2/3		0/3/24	25/1/1

### Surgical technique

**Fig. 3:**
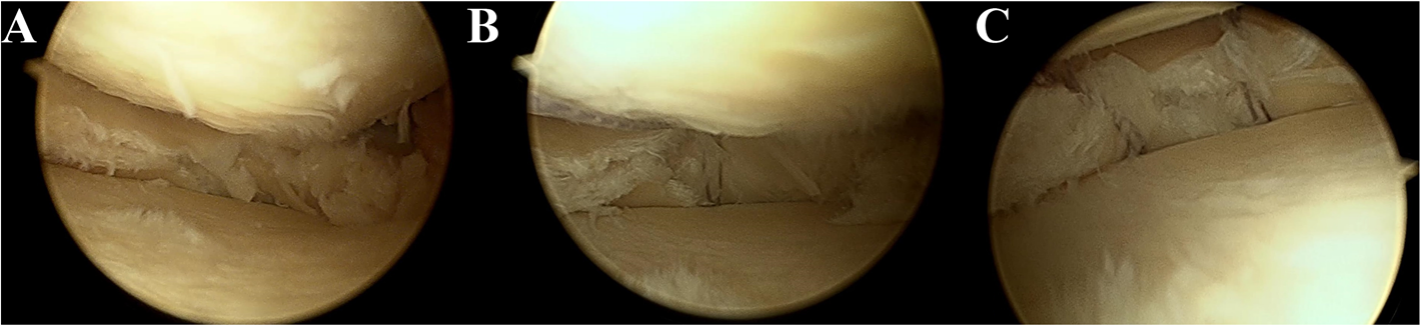
Arthroscopic repair for horizontal cleavage tears of medial meniscus. **a** Extensive horizontal tears with degeneration reached the peripheral edge of the meniscus. **b** The Omnispan needle was inserted into the tibial (inferior) surface with the first anchor, and the second anchor was placed across the horizontal tears into the femoral (superior) surface. **c** The completed repair with 5- to 10-mm intervals with the effect of a vertical (overlock) mattress suture

The knee is placed at 90° of flexion with a foot support to allow for a full range of knee motion. The knee was entered through the anterolateral and anteromedial portals. Meniscus tear and other pathologies of the knee internal structures were determined arthroscopically. It was decided to apply sutures to those with a horizontal meniscus tear with an intact meniscal ramp on the femoral and tibial joint surfaces of the meniscus. The tear edges were cleaned by shaving the irregular sections of the upper and lower meniscal parts of the tear with a shaver, punch and rasp. The meniscus repair device was entered close to the femoral meniscocapsular junction with the intact meniscus and the first suture anchor implant was placed posterior to the meniscocapsular area. Then a suture was passed from the intact meniscus close to the tibial meniscocapsular attachment point of the meniscus to the posterior of the meniscocapsular structure and placed to be vertical (overlock) and the suture knot was seated on the meniscus (Fig. 3). The Omnispan (Mitek, Norwood, MA, USA) all-inside suture instrument was used for the meniscus repairs.

### Postoperative rehabilitation

All patients started same-day postoperative patellar mobilisation exercises with isometric quadriceps exercises, and walking was permitted with two non-weight-bearing crutches. Passive knee joint exercises up to 90° were applied for 2 weeks postoperatively, and after 2 weeks, full joint movement was permitted. After the fourth week, unassisted walking with weight-bearing as tolerated was permitted and full joint movement exercises were started. In posterior meniscus tears, active knee flexion over 90°, squatting and sitting on the floor were not permitted for up to 6 weeks. At 6 months after arthroscopy, patients with no knee complaints (swelling, pulling, locking, pain and negative McMurray test) were permitted to recommence active sports and high-level activities.

### Clinical evaluation and statistical analysis

Clinical evaluation was made using the Lysholm score and Tegner activity scale preoperatively and at the final follow-up, and the differences between these two time points were analysed with Student’s *t* test. A value of *P <* 0.05 was considered statistically significant.

## Results

The patients were 16 (59.3 %) males with an mean age of 38.5 ± 11.1 years and 11 (40.7 %) females with a mean age of 40.5 ± 9.7 years. Meniscus tear repair was applied to the right knee in 15 cases and to the left in 12, medial in 21 (77.8 %) and lateral in 6 (22.2 %). The preoperative symptom of the patients and examination findings are presented in Table 2. The follow-up period of all the patients with horizontal meniscus tear repair was mean 29 months (range, 24–38). No major complications developed in any patient intraoperatively or in the early postoperative period. The IKDC Subjective Knee Evaluation Form score improved significantly from 48.5 ± 9.8 preoperatively to 90.4 ± 5.0 postoperatively (*P <* 0.0001). The Lysholm score was determined as mean 59.5 ± 12.4 preoperatively and 90.0 ± 4.7 postoperatively (*P <* 0.0001). The Tegner activity score was determined as mean 3.7 ± 1.4 preoperatively and 6.4 ± 1.6 postoperatively (*P <* 0.0001). The Cincinatti score was determined as mean 14.8 ± 5.3 preoperatively and 26.9 ± 1.6 postoperatively (*P <* 0.001) (Table 2).

**Table 2:** Knee scores

Knee scores	Preoperative	Postoperative 24 months	*P*
Subjective IKDC scores	48.5 ± 9.8	90.4 ± 5.0	0.0001
Lysholm scores	59.5 ± 12.4	90.0 ± 4.7	0.0001
Tegner activity scores	3.7 ± 1.4	6.4 ± 1.6	0.0001
Cincinnati scores	14.8 ± 5.3	26.9 ± 1.6	0.0001

According to the Kellgren-Lawrence grading on the knee radiographs, the degeneration was determined as grade 0 in 20 cases, grade 1 in 4, grade 2 in 3 and grade 3 in 0. Insufficient recovery was determined on the postoperative MRI of one patient. Insufficient recovery was determined on the postoperative MRI of one patient. According to the Barrett criteria, in that patient the McMurray test was positive and there was sensitivity in the joint line but no effusion. Second-look arthroscopic surgery is recommended for these patients, but in this case, the patient refused any further surgery.

## Discussion

This research indicates that new-generation all-inside sutures are safe and successful treatment choices for horizontal meniscus tears. In horizontal meniscus tears, repairing would be beneficial for protection of the meniscus in most of patients except only meniscectomy of an instabil inferior weak leaf-shaped part. The primary function of the meniscus is weight-bearing and transfer between the knee. Compressive load-bearing on the knee is transferred through the meniscus at 50 % in extension and at 90 % at 90° flexion [11]. The meniscus plays a significant role in the stability of the knee [12]. The meniscus provides compatibility between the incompatible femoral condyle and tibial plateau and acts as a secondary stabiliser when there is no anterior cruciate ligament [13]. The meniscus reduces the friction factor in the knee and has lubrication functions [14].

Meniscectomy is the most commonly applied method for treatment of horizontal meniscus tears [7]. In a meta-analysis, no difference was reported in a comparison of the outcomes of horizontal meniscus tears which had been repaired and those which had not been repaired [15]. However, other studies have reported that the clinical results of meniscus repair were better in cases where meniscectomy had been applied [16]. There are also studies reporting good results of repair in horizontal meniscus tears [17, 18]. Following arthroscopic repair, the anatomic shape of the meniscus is regained and it can be made properly functional. Due to importance of the meniscus for joint functions, protection is especially necessary in the young age group. In addition, meniscus repair is important to protect the thickness and width of the rim of the meniscus to preserve appropriate meniscus function in its original form, which reduces the risk of osteoarthritis. Meniscus repair is preferred particularly for patients who wish to return to a high level of activity [19, 20].

As it is accepted that horizontal meniscus tears are avascular, surgical techniques have been developed to stimulate and increase recovery. These include mechanical abrasion, fibrin clot delivery system [21], augmentation [22], gelatin hydrogels incorporating fibroblast growth factor 2 on meniscal application [23], bone marrow-stimulating technique [18] and various meniscus repair materials such as biological enhancement [24] and these have been applied in attempts to provide neovascularisation of the meniscus tissue. Ahin et al. applied bone marrow stimulation in the repair of horizontal meniscus tears and reported good results recommending this as an alternative treatment method for avascular and degenerated tears [18]. In the current study, augmentation and fibrin clot were not applied to any patient. In the patients of the current study, as there were no ACL tears or cartilage lesions, no additional surgical intervention was applied such as ACL reconstruction, microfracture, mosaicplasty or mechanical abrasion to the meniscus. Therefore, there was no agent in the environment which could have stimulated recovery. In the majority of horizontal tears, even if the meniscus recovers, the volume can be seen to be reduced. According to Madhusudhan et al., specifically, positive and negative predictive values were more favourable for clinical examination though MRI was more sensitive for meniscal injuries. The use of MRI as a supplemental tool in the management of meniscal and ligament injuries should be highly individualised by an experienced surgeon [25]. In preoperative MRI analyses, all patients had type 3 horizontal meniscus tear in this study. Patients who did not have type 1 tear or had no hyperintensity in postoperative MRI analyses were accepted to be recovered. Nevertheless only in one patient with non-healing (type 3) was continuing.

As patient age increases, there is also an increase in the rates of complex horizontal tears and cartilage lesion in the knee [26]. In the current study, as the patient age increased (>40 years), an increase was observed in complex meniscus tears, cartilage lesion in the knee and degenerative changes. In some of the patients aged over 40 years, the meniscus tear had become complex and in some patients there was insufficient meniscus tissue remaining for repair. In our clinic, partial meniscectomy is applied to these patients with horizontal complex and degenerated meniscus tear.

Terzidis et al [26] reported that of 378 young athletes with isolated meniscus tear, 22.5 % were horizontal meniscus tear. Kim et al. [27] compared the results of partial meniscectomy applied to traumatic and non-traumatic isolated horizontal meniscus tears in patients aged below 40 years and found no statistically significant difference between the two groups in respect of physical examination findings, subjective IKDC scores and Lysholm functional scores.

Following successful repair of horizontal cleavage tears, recovery has been seen, but these have been reported more with the application of open surgery techniques [17, 28, 29]. Saliman [30] reported that with the peripheral tightening meniscus suture technique for horizontal meniscus cleavage tears, the meniscus recovery was better. In the current study, when repairing the horizontal meniscus tears, suturing was applied using the all-inside suture to the separated meniscus layers in such a way as to create peripheral compression.

Excellent recovery rates of 83 %–91 % have been reported in meniscus repair made with all-inside meniscus devices [31, 32]. Repairs made with second-generation all-inside meniscus repair instruments have shown it to be a good, long-lasting and reliable method with survival rates over 5 years reported of 84 % [32]. In the current study, the results of the repair of horizontal meniscus tear with new-generation all-inside sutures on selected patients were good and evaluation of the physical examination findings, subjective IKDC scores, Lysholm functional scores and Tegner activity scores showed it to be a successful treatment option.

The major limitation of this study was that it was retrospective. A second limitation is that no comparison was made between the patients to whom all-inside meniscus repair was applied and those who underwent meniscectomy for horizontal meniscus tear. Neither was any comparison made with non-operated horizontal tears. With a greater number of patients and the application of second-look arthroscopy, the extent of the meniscus recovery could have been determined.

## Conclusions

The results of this study have shown that arthroscopic all-inside horizontal meniscus repair is a successful option in the preservation of a torn meniscus. The physical examination and functional results were good. With careful selection of the patients and the horizontal meniscus tears, the success of the meniscus repairs increases. Repair can be recommended for all horizontal meniscus tears which can be repaired arthroscopically.

## Abbreviations

IKDC: International Knee Documentation Committee
MRI: Magnetic resonance imaging
